# Epidural haematoma following vertebroplasty for osteoporosis compression fracture: A case report

**DOI:** 10.1016/j.ijscr.2025.111727

**Published:** 2025-07-30

**Authors:** Yongsheng Ye, Fangyue Deng, Yonghong Feng, Linfeng Luo, Henian Li, Shabin Zhuang

**Affiliations:** Department of Orthopedics, Dongguan Hospital of Traditional Chinese Medicine, Dongcheng District, Dongguan City, Guangdong Province 523000, China

**Keywords:** Osteoporotic vertebral compression fracture, Percutaneous kyphoplasty, Percutaneous vertebroplasty, Spinal epidural haematoma, Rare complication, Case report

## Abstract

**Introduction:**

Percutaneous vertebroplasty (PVP) is a minimally invasive procedure commonly used to alleviate pain and stabilise vertebral compression fractures caused by osteoporosis. Although generally safe, the procedure carries the risk of rare complications, such as epidural haematomas.

**Presentation of case:**

We present the case of a 67-year-old woman who underwent PVP for an abnormal pedicle structure associated with an osteoporotic compression fracture that led to the subsequently development of an epidural haematoma. The patient presented with severe back pain following a fall at home. Imaging revealed a T12 vertebral compression fracture with marked collapse and posterior wall involvement. The pain and functional impairment persisted despite conservative management. After polymethylmethacrylate injection into the fractured vertebrae, the patient reported unbearable lower back pain and bilateral lower limb weakness, which progressed to paraplegia.

**Discussion:**

Magnetic resonance imaging of the spine revealed bilateral epidural haematomas at T11–T12, causing significant spinal cord compression. Urgent surgical decompression was performed to relieve nerve compression. Neurological symptoms improved gradually.

**Conclusion:**

This case highlights the importance of early diagnosis and management of the complications of PVP, including epidural haematoma.

## Highlights

• PVP involves the injection of a biomaterial to manage vertebral fractures.
• Epidural haematoma may be caused by the puncture of the epidural venous plexus.
• Preoperative CT and MRI analysis can reduce the risk of epidural haematoma after PVP.

## 1 Introduction

Percutaneous vertebroplasty (PVP) is a widely used procedure for managing painful osteoporotic vertebral compression fractures (OVCF), which can provide significant pain relief and improve function. However, PVP is associated with potential complications, including infection, neurological injury, bowel or bladder dysfunction, bleeding, rib fracture, cement leakage, adjacent vertebral fracture, and death due to pulmonary embolism [[1], [2], [3]]. Acute spinal epidural haematoma (SEH) is a rare but potentially severe complication that warrants attention in clinical practice. Herein, we report a case of pedicle structure abnormality that developed into SEH following PVP. This case has been reported in line with the SCARE criteria [1].

## 2 Presentation of case

**Fig. 1:**
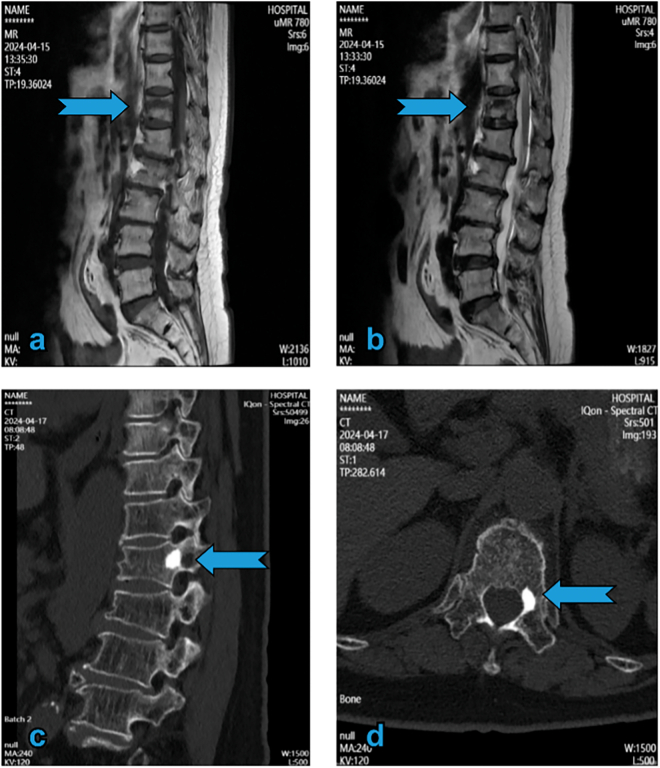
MRI of T1 and T2 sequences and preoperative CT. (a and b) Revealing vertebral compression fracture with hypointensity in T1 and hyperintensity in T2 (arrow). Preoperative CT (c) showing sclerosis lesions in the inner wall of the left pedicle in T12 (arrow). MRI: magnetic resonance imaging; CT: computed tomography.

A 67-year-old woman presented to our clinic with severe back pain after a fall at home. Imaging revealed a T12 vertebral compression fracture with significant collapse and posterior wall involvement (Fig. 1a, b). Pain and functional impairment persisted despite conservative management. Therefore, she was deemed eligible for PVP, which was conducted by a board-certified spinal surgeon in the hospital's main operating room. Polymethylmethacrylate was injected into the fractured vertebrae under fluoroscopic guidance. No cement leaked into the spinal canal (Fig. 3c, d).

**Fig. 2:**
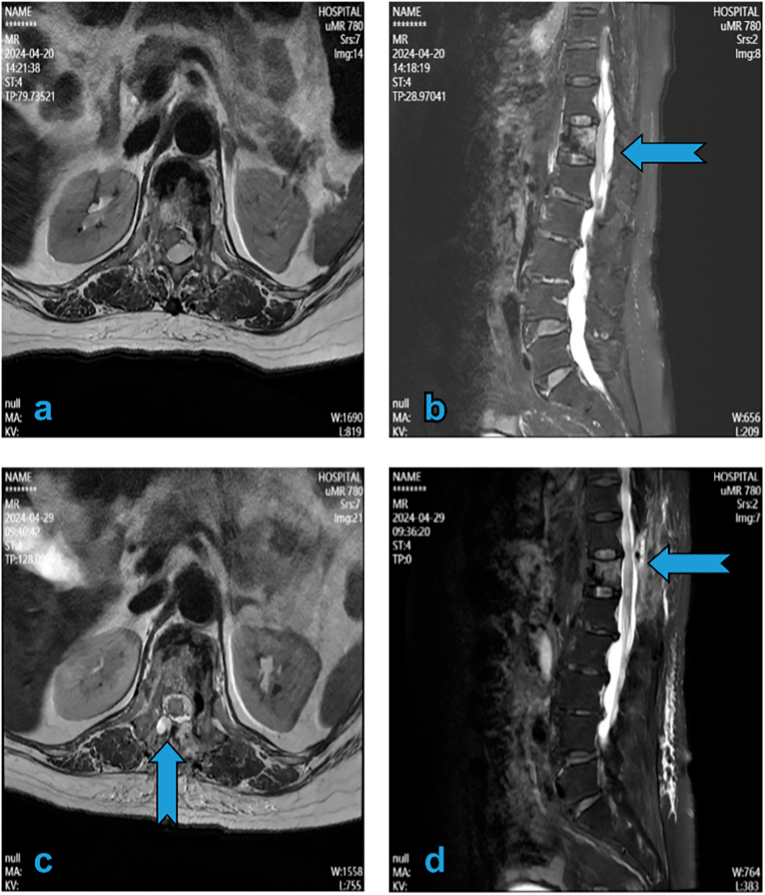
T2 vertebra MRI scan. (a and b) Hyper-acute epidural hematoma over T11 extending to the L1 vertebrae. Axial images showing epidural hematoma compressing the spinal cord (arrow). (c and d) MRI after hemilaminectomy decompression and hematoma removal showing resolution of both SEH and nerve compression (arrow). MRI: magnetic resonance imaging; SEH: spinal epidural haematoma.

Postoperatively, the patient exhibited improved mobility and no neurological deficits; nonetheless, she continued to have severe lower back pain. Approximately 1 h postoperatively, the patient complained of cold lower limbs. Skin examination revealed stable normal findings on palpation, and the dorsalis pedis artery had a normal pulse. She subsequently developed bilateral lower limb weakness. The neurological condition worsened, and she developed paraplegia. Magnetic resonance imaging (MRI) of the spine revealed bilateral SEH at T11–T12 that caused spinal cord compression (Fig. 2a, b).

Urgent surgical decompression was performed given the neurological deterioration and spinal cord compression due to SEH. Decompression was achieved via laminectomy without requiring supplemental spinal instrumentation (Fig. 4a, b). Intraoperatively, clotted blood was observed in the epidural space, exerting pressure on the spinal cord. After decompression, MRI revealed that SEH had been eliminated, and that the nerve was no longer compressed (Fig. 2c, d). Neurological symptoms improved gradually. Two weeks postoperatively, lower limb strength returned to grade IV, and skin sensation was normalised.

## 3 Discussion

PVP and percutaneous kyphoplasty (PKP) are well-established treatments for OVCF to alleviate pain and improve quality of life [2,5]. However, these procedures can cause complications, including pedicular fractures, nerve root injuries, and spinal cord lesions [3,4]. Spinal subdural haematoma (SSDH) and SEH are rare complications that usually occur a few hours after completion of PVP or PKP [6,7]. SSDHs can occur in the lower thoracic region, typically affecting two to five segments [7]. SSDHs are typically located anterior to the spinal cord, whereas SEHs are usually located posterior to the spinal cord [8]. To the best of our knowledge, a few SSDH cases following PVP have been reported, whereas SEH following PVP has been reported less frequently [6,7,[9], [10], [11], [12], [13], [14]].

SEHs can be classified as idiopathic, iatrogenic, traumatic, or coagulopathic. The analysis of over 1000 cases of SEH revealed that iatrogenic causes, such as coagulopathy or spinal puncture, accounted for 18 % of cases, while non-iatrogenic causes, such as genetic or metabolic coagulopathy, trauma, and pregnancy, comprised 29 %. The aetiology was multifactorial in 11.1 % of cases [15].

The aetiology of SEH is unclear however, it might include surgical iatrogenic factors, venous congestion, comorbidities, and anticoagulant therapy. Various iatrogenic causes might lead to SEH, including vascular injury during needle insertion, venous congestion caused by bone cement extravasation, and rupture of the epidural venous plexus. Cosar et al [11] suggested that spinal SEH can develop following a puncture of the spinal dura mater, allowing venous blood to infiltrate the subdural space post-trauma. Wang et al. emphasized that SEH after PKP resulted from damaged blood vessels caused by intraoperative puncture, which occurs when the rupture of the inner wall of the pedicle causes extravasation of blood within the cancellous bone, leading to blood flow into the epidural space [14].

Another potential cause of SEH is venous congestion due to bone cement leakage, which can increase pressure in the paravertebral veins, including the radiculomedullary veins, leading to SSDH and rupture of the epidural venous plexus [16].

Patients with underlying diseases, such as hypertension, coagulopathy, and thrombocytopenia, have an increased risk of bleeding. Hypertension before surgery can lead to spontaneous SEH [10]. Vascular malformation is also considered one of the causes of spontaneous SEH.

The pre- or postoperative use of anticoagulants might affect coagulation function and increase the risk of haematoma formation. Brelie et al. reported that SEH after PVP was due to coagulopathy induced by the long-term use of oral aspirin [17]. Zou reported that, in the absence of intraspinal cement leakage, the combination of pedicle puncture and the use of anticoagulants might cause SEH after PKP [14].

**Fig. 3:**
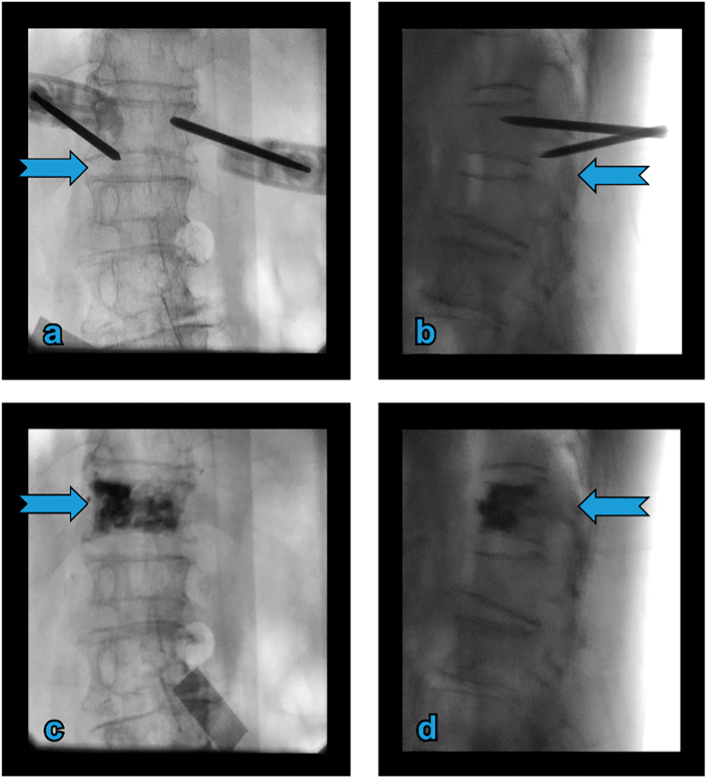
Intraoperative fluoroscopy. (a and b) The left puncture needle accidentally penetrating the intervertebral foramen. (c and d) Anteroposterior view of the lumbar radiograph showing no evident intraspinal cement leakage.

Our patient had normal haemostatic parameters (preoperative international normalised ratio, 1.0; platelet count, 200 × 10^9^/L), received no anticoagulants treatment, and normal baseline health metrics (blood pressure, 120/75 mm Hg; HbA1c, 5.2 %). We analysed the pathogenesis of the present case based on previous hypotheses. Direct puncture injury to the spinal cord during surgery was not likely as the patient had intact sensory and motor functions in the lower limbs after surgery. However, examination of the intraoperative puncture revealed that the left pedicle area was blocked by sclerosis (Fig. 1c, d), which prevented the puncture needle from entering the vertebrae via the transpedicular approach. The puncture needle inadvertently entered the nerve root exit area during needle adjustment, causing vessel injury (Fig. 3a, b).

**Fig. 4:**
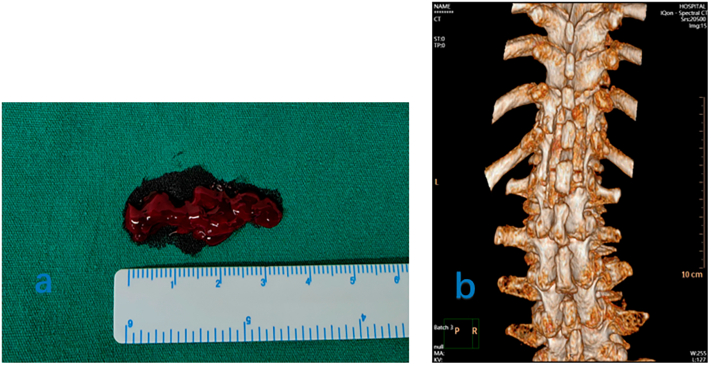
Intraoperative imaging and postoperative CT findings. (a) Clotted blood is removed from the epidural space intraoperatively. (b) CT showing the extent of lamina decompression postoperatively. CT: computed tomography.

In our patient, the haematoma was located in the epidural space. The loose connective tissue in this space allows blood extravasation. The anterior and posterior spaces can be affected. The anterior space is located between the posterior part of the vertebral body and the dura mater, whereas the posterior space is located between the lamina and the dura mater. Bone cement leakage or damage to the posterior wall of the vertebral body can occur in the anterior space, resulting in bleeding. In the posterior space, the puncture needle can injure the epidural venous plexus or muscle vessels.

Preoperative computed tomography and other imaging data should be evaluated to identify physiological or structural abnormalities and to plan surgery. If an abnormal pedicle structure or puncture path is detected preoperatively, alternative puncture paths, such as those via the costovertebral joint or a unilateral transpedicular approach, should be considered. These alternatives decrease the risk of pedicle fracture, medial transgression of the pedicle, transgression into the spinal canal, nerve injury, cement extravasation along the cannula tract, and SEH [18].

Preventing SEH after PVP requires a continuous effort throughout the perioperative period. Improving coagulation and adjusting basic conditions before the operation are essential. For this purpose, three measures are recommended. The first includes assessing coagulation function. Preoperative tests are routinely performed to quantify four coagulation indicators and platelet count. The international normalised ratio should be ≤1.5, platelet count ≥100 × 10^9^/L, and anticoagulant drugs such as aspirin and warfarin should be discontinued before the operation. The second measure includes controlling underlying diseases, such as hypertension. Before the operation, blood pressure should be maintained below 140/90 mm Hg to reduce the risk of bleeding during puncture. The third measure includes conducting preoperative CT and MRI scans to assess the integrity of the posterior vertebral wall and the condition of the bone cortex and to predict injury to blood vessels during puncture.

Management includes emergency surgical decompression and conservative treatment. In patients with spinal cord compression (such as ASIA grade ≤ C) and progressive neurological dysfunction, laminectomy is performed for decompression and hematoma removal, whereas in patients with spinal cord compression (ASIA grade ≥D) conservative treatment is selected.

## 4 Conclusion

Although PVP is considered safe and effective for managing compression fractures, clinicians should remain vigilant for potentially severe complications, such as SEH. Prompt recognition and early surgical intervention are crucial for optimising patient outcomes. Further studies are necessary to identify risk factors and develop preventive strategies for PVP.

## CRediT authorship contribution statement

Y.S.Y. and F.Y.D. drafted the manuscript. Y.H.F. and H.N.L. designed the study. L.F.L. collected data. S.B.Z. performed all surgical procedures. All authors read and approved the final manuscript.

## Consent

Written informed consent was obtained from the patient for publication of this case report and accompanying images.

## Ethical approval

This study was approved by the Ethics Committee of our institution, and written informed consent was obtained from the patient.

## Guarantor

Shabin Zhuang.

## Research registration number

Not applicable.

## Funding

This study was supported by the Administration of Traditional Chinese Medicine of Guangdong Province (Grant No. 20251420), the Famous Traditional Chinese Medicine Studio of Dongguan Hospital of Traditional Chinese Medicine (Grant No.50202505002), and the Dongguan Social Development Science and Technology Project (Grant No. 20221800906042).

## Declaration of competing interest

The authors declare no competing interests.

## Data Availability

The data that support the findings of this study are available from the corresponding author upon reasonable request.
